# The Interplay of Vocabulary, Working Memory, and Math Anxiety in Predicting Early Math Performance

**DOI:** 10.3390/jintelligence13100125

**Published:** 2025-09-29

**Authors:** Roberto A. Ferreira, Cristina Rodríguez, Bárbara Guzmán, Felipe Sepúlveda, Christian Peake

**Affiliations:** 1 Facultad de Ciencias de la Educación, Universidad de Talca, Linares 3582979, Chile; 2 Millennium Nucleus for the Science of Learning (MiNSoL), Linares 3582979, Chile; 3 Facultad de Educación, Universidad Católica de la Santísima Concepción, Concepción 4090541, Chile; 4 Facultad de Educación, Universidad Diego Portales, Santiago 8370067, Chile

**Keywords:** mathematical performance, early childhood, math anxiety, working memory, math vocabulary, language development, early numeracy, structural equation modelling, primary education, cognitive-affective factors

## Abstract

Mathematical performance in early education is influenced by a complex interplay of cognitive and affective factors, including language skills, working memory, and anxiety. This study investigated whether working memory and math anxiety, in both explicit numerical situations (ENS) and general classroom situations (GCS), mediate the relationship between general and math-specific vocabulary and math performance in a sample of 467 second-grade students in Chile. Structural equation modelling was employed to test a dual-pathway model in which both working memory and math anxiety served as mediators between vocabulary knowledge and math performance. Results indicated that both general and math-specific vocabulary positively predicted working memory and negatively predicted math anxiety in ENS. In turn, working memory and ENS significantly predicted math outcomes, whereas GCS was not a significant predictor. Indirect effects supported a dual mediation structure, with vocabulary influencing math performance through both cognitive and affective mechanisms. Math-specific vocabulary exerted a slightly stronger total effect than general vocabulary, consistent with its closer alignment to the semantic demands of mathematical tasks. These findings suggest that vocabulary supports early mathematical learning not only by enhancing cognitive processing capacity but also by reducing anxiety in task-specific contexts.

## 1. Introduction

Early mathematical skills are widely recognized as some of the most robust predictors of later academic achievement, often surpassing early reading abilities in their predictive value (Duncan et al. 2007; Watts et al. 2014). Mathematics and language are fundamentally interconnected cognitive domains, each supporting and enhancing the development of the other (e.g., Child et al. 2019; Korpipää et al. 2017). This interdependence is particularly salient during the early years of formal education, when children begin to engage with more cognitively demanding tasks (Peng et al. 2019). A recent meta-analysis by Peng and Lin (2020) quantified the strength of this association, reporting a moderate correlation (r = 0.42) between language and mathematics, with more advanced language abilities showing a stronger association with mathematical competence. Longitudinal evidence further underscores this interdependence, demonstrating that early proficiency in both language and mathematics reliably predicts later academic outcomes (e.g., Purpura et al. 2017).

In addition to the moderate association between language and mathematics, cognitive factors such as working memory have also been shown to play a central role in both domains. Working memory, the capacity to temporarily hold and manipulate information, is essential not only for language comprehension but also for executing a range of mathematical tasks, including calculation and problem-solving (Alloway and Alloway 2010; Korpipää et al. 2017). These overlapping demands raise important questions regarding the potential mediating role of working memory in the association between language proficiency, particularly vocabulary, and mathematical performance.

While cognitive factors such as language and working memory are essential for mathematical learning, affective factors, particularly math anxiety, also play a critical role in shaping math performance. Math anxiety—conceptualized as a state of tension, apprehension, and physiological arousal in response to numerical demands (Mammarella et al. 2023)—has been robustly linked to lower mathematics achievement across the lifespan (Barroso et al. 2021; Caviola et al. 2022). Crucially, this association is already evident in the early years of formal education. Longitudinal research demonstrates that math anxiety can emerge in primary school and predict subsequent arithmetic underperformance (Pellizzoni et al. 2022; Sorvo et al. 2019). Furthermore, recent studies suggest that even young children exhibit measurable levels of math anxiety, with variations in intensity and underlying causes (Szczygieł and Pieronkiewicz 2022).

Given its early onset and consistent association with underperformance, it is critical to understand how maths anxiety interacts with cognitive mechanisms, particularly working memory. Maths anxiety is known to consume cognitive resources by occupying working memory with intrusive, self-referential thoughts, thereby reducing the mental capacity available for problem-solving and vocabulary retrieval (Ashcraft and Kirk 2001; Brunyé et al. 2013). This disruption is especially detrimental during tasks that require the integration of linguistic and numerical processing, such as interpreting verbal problem statements or applying maths-specific terminology. Recent findings by (Zhang et al. 2024) further clarify that factors like time pressure, task difficulty, and fear of failure amplify anxiety, thereby exacerbating its interference with working memory and impairing mathematical performance.

This study investigates whether working memory and two dimensions of maths anxiety—explicit numerical situations and general classroom-based situations—mediate the relationship between general and maths-specific vocabulary and mathematical performance. By modelling these cognitive and affective mechanisms simultaneously, we aim to elucidate how language, memory, and anxiety interact in the context of early mathematical learning.

### 1.1. The Relationship Between Mathematics and Language, Particularly Vocabulary

Mathematics and language are deeply interconnected cognitive domains, with each domain enriching the other’s comprehension and proficiency. This relationship is evident in how mathematical concepts are communicated and conceptualized through linguistic structures, as language provides the symbols and structures necessary for mathematical reasoning and problem-solving, ranging from simple counting to complex algebraic operations (Macbeth 2012). Linguistic abilities, particularly vocabulary breadth and syntactic comprehension, have been shown to play a pivotal role in mathematical development, especially during the early years of schooling (LeFevre et al. 2010; Purpura and Logan 2015).

Most empirical evidence examining the relationship between mathematics and language has conceptualized language primarily in terms of vocabulary, distinguishing between general vocabulary and mathematics-specific vocabulary. General vocabulary, a fundamental aspect of language development, provides a robust foundation not only for overall academic success but also for enhancing mathematical capabilities. The interplay between general vocabulary and mathematical performance has been extensively studied, consistently revealing a positive correlation (Purpura and Ganley 2014; Riccomini et al. 2015). A well-developed general vocabulary equips children with the linguistic resources needed to interpret word problems, understand instructions, and make sense of mathematical contexts, thereby facilitating their conceptual grasp of mathematical ideas (Peng et al. 2019). This relationship appears particularly strong in early education settings, where more advanced verbal skills have been associated with higher mathematical proficiency (Peng and Lin 2020). In addition to specialized terms, everyday language, including words like “más” (more), “menos” (fewer), and “juntos” (together), facilitates mathematical reasoning by providing intuitive linguistic cues for quantitative concepts (Purpura et al. 2017). Syntactic comprehension is equally critical, as the structure of mathematical word problems often relies on complex sentence constructions to convey relationships (LeFevre et al. 2010). In multilingual settings, these linguistic elements may pose additional challenges, though our sample focused on monolingual Spanish speakers.

In contrast, mathematics-specific vocabulary comprises terms uniquely tied to numerical and mathematical contexts, such as “sum”, “difference”, “denominator”, or “equation”. These specialized terms provide the linguistic scaffolding necessary for engaging with mathematical concepts and problems effectively. Importantly, mathematics vocabulary has been found to be a unique and robust predictor of mathematical achievement, over and above general vocabulary and executive functions (Kung et al. 2019; Purpura and Logan 2015). For instance, Toll and Van Luit (2014) showed that maths-specific vocabulary mediated the association between general language skills and mathematical ability, suggesting that acquiring precise mathematical terminology is a critical developmental milestone for numeracy. As students encounter more complex mathematical problems, this vocabulary becomes increasingly vital for successful performance. Accurate interpretation and use of terms like “quotient” or “variable” can determine whether a student solves a word problem correctly or misinterprets its structure entirely (Peng et al. 2019). For example, understanding and correctly using terms like “difference” or “product” can significantly affect a student’s ability to interpret and solve word problems or engage in algebraic thinking.

In sum, both general and mathematics-specific vocabularies function as critical linguistic tools for navigating the demands of mathematics. While general vocabulary supports the comprehension of problem contexts and verbal instructions, mathematics-specific vocabulary enables precise reasoning, symbolic representation, and the manipulation of abstract concepts. As such, vocabulary development in both domains is essential for fostering mathematical competence throughout early schooling.

### 1.2. Intersection of Vocabulary, Working Memory, Math Anxiety, and Math Performance

The impact of vocabulary on mathematical performance does not occur in isolation; it emerges from a dynamic interplay with other cognitive and affective mechanisms, most notably working memory and math anxiety. Studies have shown a reciprocal relationship between vocabulary and working memory: on the one hand, a robust vocabulary enhances encoding and retrieval processes by offering efficient linguistic scaffolds for cognitive tasks (Ashcraft and Kirk 2001; Gathercole and Baddeley 1993); on the other hand, individuals with strong working memory capacity are better equipped to acquire, store, and manipulate new vocabulary, supporting richer semantic knowledge (Daneman and Green 1986; Vallée-Tourangeau et al. 2016). This bidirectional connection has been corroborated by longitudinal and experimental research, which shows that vocabulary depth can reinforce phonological working memory capabilities and vice versa (Language and Reading Research Consortium (LARRC) et al. 2019; Speciale et al. 2004). Such findings suggest that these domains form a feedback loop in which language and memory develop interdependently.

Importantly, this interdependence directly informs mathematical learning. Children with larger vocabularies often show stronger performance in mathematics, as lexical knowledge supports both the understanding of quantitative concepts and the verbal articulation of mathematical reasoning (Kucian et al. 2018; Ruijia et al. 2022). Vocabulary aids not only in decoding mathematical problems but also in comprehending instructions, interpreting task demands, and applying arithmetic procedures accurately (Kiss and Vukovic 2020; Passolunghi et al. 2016). Thus, vocabulary proficiency may serve as a foundational gateway to higher-order math cognition.

Working memory, in turn, is widely recognized as a central predictor of math performance. It enables learners to retain, update, and manipulate information during problem solving, especially in tasks that require multiple steps or involve abstract reasoning (Bull and Lee 2014; Fuchs et al. 2005). Higher working memory capacity has been linked to greater fluency in arithmetic operations and more accurate retrieval of math facts, while limited capacity may hinder performance, particularly under cognitively demanding conditions (Ganley and McGraw 2016; Passolunghi et al. 2016). Moreover, research indicates that the effectiveness of language-based instruction in mathematics depends, at least in part, on students’ working memory abilities, with those having lower capacity benefiting more from conceptual scaffolding (Fuchs et al. 2016).

However, math anxiety introduces a significant constraint on this otherwise productive interaction between vocabulary and working memory. Defined as a state of tension and apprehension in response to mathematical demands (Mammarella et al. 2023), math anxiety has been shown to disrupt working memory functioning by consuming cognitive resources that would otherwise support problem solving (Ashcraft and Kirk 2001; Wu et al. 2017). Individuals experiencing high levels of math anxiety are more likely to exhibit intrusive thoughts, reduced attentional control, and impaired retrieval of relevant information, especially under time pressure or task complexity (Daker et al. 2021). Neuroimaging evidence further supports this view, showing that math anxiety activates brain regions associated with error monitoring and cognitive control, leading to delayed response times and reduced accuracy in working memory-intensive tasks (Hunt and Sandhu 2017; Pelegrina et al. 2020; Rose et al. 2023).

As a result, students with strong vocabulary and cognitive potential may still underperform in mathematics if they experience elevated math anxiety. In this context, math anxiety may function as a mediating mechanism that undermines the benefits of cognitive resources. Daker et al. (2021) found that students with adequate math knowledge still demonstrated poor performance under anxiety-inducing conditions, suggesting that emotional interference can override skill-based advantages. Similarly, (Pizzie and Kraemer 2023) argue that math anxiety interacts with both working memory and vocabulary, creating a complex cognitive-affective barrier to math achievement.

Taken together, these findings support a conceptual model in which both working memory and math anxiety mediate the relationship between vocabulary, particularly math-specific vocabulary, and math performance. While vocabulary supports the comprehension and execution of mathematical tasks via its connections to working memory, math anxiety disrupts these processes by diverting attention, increasing cognitive load, and impairing memory retrieval (Braham and Libertus 2018; Justicia-Galiano et al. 2017). In this framework, working memory serves as a facilitator and math anxiety as an inhibitor, each shaping how effectively vocabulary knowledge is translated into mathematical competence.

### 1.3. The Current Study

In the present study, math anxiety is assessed as a multidimensional construct comprising two latent components: anxiety in Explicit Numerical Situations (ENS) and anxiety in General Classroom Situations (GCS), using the Child Mathematics Anxiety Questionnaire (Ramirez et al. 2013). This distinction enables a more precise investigation of how math anxiety operates across different learning contexts, particularly whether anxiety directly tied to numerical content (ENS) exerts a stronger mediating effect on math performance than more generalized school-related anxiety (GCS). While studies like Peng and Lin (2020) and Hornburg et al. (2024) have explored vocabulary and working memory in relation to math performance, few have investigated the dual mediation of working memory and math anxiety, particularly in Spanish-speaking children. Susperreguy et al. (2024) examined math-specific vocabulary in Chilean students but did not incorporate math anxiety or mediation analyses. Our study uniquely integrates these cognitive and affective factors in a large, diverse sample, addressing a critical gap in early mathematical learning research.

We address the following research questions:

1. What are the relationships between general vocabulary, math-specific vocabulary, math anxiety, and math performance in Chilean second-grade students?
2. Do working memory and math anxiety mediate the relationships between vocabulary (general and math-specific) and math performance?

Based on the reviewed literature, we hypothesize that:

- Both general and math-specific vocabulary will be positively associated with math performance.
- Math anxiety will be negatively associated with math performance.
- Working memory will mediate the relationship between both types of vocabulary and math performance, such that better vocabulary knowledge enhances working memory, which in turn supports higher math performance.
- Math anxiety (ENS) will mediate the relationship between both types of vocabulary and math performance, such that better vocabulary knowledge reduces anxiety, which in turn improves performance.
- The indirect effects of math-specific vocabulary on math performance will be stronger than those of general vocabulary, due to its greater relevance for mathematical content and procedures.

## 2. Materials and Methods

The present study forms part of a broader longitudinal research project that tracks the cognitive, linguistic, and academic development of children from prekindergarten through third grade. In this phase of the project, data were collected during the second grade to examine the interplay between vocabulary (general and math-specific), working memory, math anxiety, and math performance. To do so, children completed a comprehensive battery of individual assessments designed to evaluate multiple domains, including non-verbal intelligence, receptive and expressive vocabulary, spatial and numerical working memory, and early mathematical abilities. The principal outcome measure was performance on Test of Early Mathematics Ability (TEMA-3) adapted for Spanish speaking children (Ginsburg et al. 2007).

### 2.1. Participants

The initial sample comprised 503 second-grade children from 16 schools in the central-south region of Chile, representing a broad and demographically diverse group. Schools were selected to include public (7.49%, *n* = 35, mean SVI = 92.60), private subsidized (81.80%, n = 382, mean SVI = 70.20), and private institutions (10.71%, *n* = 50, mean SVI = 0), reflecting varied socioeconomic contexts as indicated by the Social Vulnerability Index (SVI; overall M = 64.33, SD = 25.53, range = [0, 95.89]), a measure of school-level socioeconomic disadvantage (Ministerio de Educación de Chile 2025). Participants were monolingual Spanish speakers educated in Spanish, the primary language of instruction in Chilean schools. After excluding 36 cases (7.2%) due to incomplete data for structural equation modelling, the final analytic sample included 467 children (230 boys, 237 girls; mean age = 95.9 months, SD = 3.5). Participants were originally recruited through school partnerships during their prekindergarten year, and informed consent was obtained from parents or legal guardians prior to participation. The study received ethical approval from the Ethics Committee of the Universidad Católica del Maule and was conducted in accordance with national and institutional ethical standards.

### 2.2. Measures and Instruments

#### 2.2.1. Semantic Fluency

A semantic fluency task was chosen to assess general vocabulary due to its established validity in measuring expressive language skills in young children (Olmos-Villaseñor et al. 2023). This task captures the breadth of lexical knowledge, which supports mathematical comprehension (Purpura and Ganley 2014), and is suitable for second graders due to its engaging format. It has traditionally been employed in neuropsychology as a screening tool to assess cognitive decline in conditions such as Alzheimer’s disease and other forms of dementia (Lin and Chih 2023; Olmos-Villaseñor et al. 2023). It has also been widely used in psycholinguistics and educational research to evaluate vocabulary production in both children and adults, where it is formally known as lexical availability (Agustin-Llach 2022; Ferreira et al. 2019). However, to the best of our knowledge, it has never been utilized as a measure of general vocabulary in the context of mathematical cognition.

In this task, participants were asked to verbally generate as many words as possible from two semantic categories—animals and food & drink—within a one-minute timeframe for each category. Participants were informed that their responses should consist only of unique words from the specified category, and any repetitions were not counted. Instructions were displayed on a computer screen, and an examiner recorded the words in the order they were spoken. The session was also audio-recorded to ensure accuracy in capturing the participant’s responses. If the participant remained silent for 15 s, the examiner would repeat the instructions and encourage further word production. Repeated words were excluded from the final count. The task required only verbal responses, and no written or gestural communication was allowed. Split-half reliability for the semantic fluency task was assessed by considering the scores for animals and food & drink and calculating the correlation between the two categories, resulting in a reliability coefficient of r = 0.54. The Spearman–Brown corrected reliability for the entire task was found to be 0.70, indicating adequate internal consistency or even strong in view that these categories are two distinct domains.

#### 2.2.2. Auditory Lexical Decision

The auditory lexical decision task was selected to assess math-specific vocabulary due to its sensitivity to rapid word recognition, a foundational aspect of vocabulary knowledge (Duchon et al. 2013). While recognition does not guarantee full comprehension of mathematical terms, it indicates familiarity with the lexical items critical for math tasks. If children recognize terms without understanding their meanings, the relationship between math-specific vocabulary and performance may be underestimated, potentially strengthening the observed effects with deeper comprehension measures.

We began by extracting mathematical vocabulary from Chilean textbooks for second, third, and fourth grades, identifying 437 unique words present across all textbooks for these years. Seven judges were then tasked with classifying these words into three categories: math-specific terms, math-specific terms used outside mathematics, and general vocabulary. The inter-rater reliability was moderate to substantial, with a Fleiss’ kappa of 0.56 and an intraclass correlation coefficient of 0.74. From this list, we selected only the words the judges classified as either math-specific terms or math-specific terms also used outside the field of mathematics, resulting in 141 words. We obtained the logarithmic frequency of these words from the EsPal database (Duchon et al. 2013), with a mean log frequency of 0.92 (SD = 0.70, range = 0.004–3.28).

To ensure a representative sampling across frequency bands, we divided the words into deciles based on their log frequencies, ranging from the highest to the lowest frequencies. From each decile, we randomly selected three words, ensuring that no two words shared the same root (e.g., “count” and “counting”). This process yielded a final selection of 30 words with a mean log frequency of 0.91 (SD = 0.68, range = 0.004–2.53). Additionally, we created 16 pseudowords as fillers. Given that many of the mathematical terms were unfamiliar to the participants, it was unnecessary to have an equal number of fillers and real words. This differs from typical lexical decision tasks, where participants are expected to recognize the majority of the real words, and an equal balance is maintained.

The task involved seating participants comfortably at a computer, where they were instructed to listen to audio recordings of the 30 real mathematical terms and 16 pseudowords. Participants responded by pressing a button with a “√” sign on the screen for real words or a button with an “X” sign for pseudowords, with a response window of 3 s before the next word was automatically presented. The task began with a practice session where children familiarized themselves with the task requirements followed by the actual evaluation phase. Responses were evaluated based on accuracy for correct responses only. The split-half reliability of the lexical decision task was assessed using the correlation between the two halves of the test. The correlation between the two halves was r = 0.55. Applying the Spearman–Brown correction, the split-half reliability was 0.71, which indicates that the auditory lexical decision task has an acceptable level of internal consistency.

#### 2.2.3. Math Performance

Math performance was assessed using the Test of Early Mathematics Ability, Third Edition (TEMA-3), adapted for Spanish-speaking children (Ginsburg et al. 2007). TEMA-3 was chosen over alternatives like the Woodcock-Muñoz Battery due to its comprehensive coverage of early math skills (number facts, calculation, and concepts) (Ginsburg and Baroody 2003) and established validity in Spanish-speaking populations. The test was administered individually in Spanish by trained examiners, following standardized procedures to ensure accessibility for monolingual Spanish speakers.

The tasks used in this study included number facts (knowledge of basic arithmetic facts, such as addition, subtraction, and multiplication facts, without the need for concrete objects or counting), written calculations (ability to perform arithmetic operations including addition, subtraction, multiplication, and division using written symbols and algorithms), and calculation concepts (understanding of mathematical concepts and principles underlying arithmetic operation).

Participants completed the assessment in sessions lasting 30–45 min. Standardized scores were computed from the raw data, with scores exceeding two standard deviations below the mean excluded from the analysis. The average standardized score was 6.34 (SD = 4.077), with scores ranging from 1 to 16. The internal consistency reliability of the three-item scale was evaluated using Cronbach’s Alpha, which indicated a high level of internal consistency (α = 0.86). A confirmatory factor analysis (CFA) was conducted to assess the fit of the hypothesized model. The results indicated an excellent fit to the data, with both the Comparative Fit Index (CFI) and the Tucker–Lewis Index (TLI) equal to 1.000, and minimal residuals as indicated by the Root Mean Square Error of Approximation (RMSEA) and the Standardized Root Mean Square Residual (SRMR), both at 0.000.

#### 2.2.4. Spatial Working Memory

We utilized a computerized adaptation of the Corsi block-tapping (Milner 1971) to measure spatial working memory. This task was performed on a screen displaying nine squares that lit up in a specific order. Participants were first instructed to touch the squares in the same sequence as they lit up, starting with sequences of two and progressing to sequences of five squares. After completing these sequences, the task’s second part required participants to touch the squares in the reverse order of illumination. Each participant went through five sequences in the original order and five in the reverse order. The evaluator’s role was to ensure that participants understood the instructions clearly, starting with a practice session and providing feedback such as “Great job, now move on to the next one!” if the sequences were correctly followed. If a participant failed to follow the sequence correctly, the instructions were repeated up to three times. Persistent errors led to the termination of the task. The reliability of this task was evaluated using Cronbach’s alpha based on tetrachoric correlations due to the dichotomous nature of the items. The analysis indicated excellent internal consistency (α = 0.91).

#### 2.2.5. Numerical Working Memory

The Numerical Working Memory task, adapted from Case et al. (1982) for Spanish-speaking children (Rodríguez et al. 2021), required participants to count and recall sequences of yellow dots on cards displaying a mix of blue (distracter) and yellow (target) dots, with sequences increasing from two to five cards. This adaptation reversed the original colours (green targets, yellow distracters) for better visual distinction, replaced verbal recall with selection from a list of numbers (0–9) on the screen to minimize verbal demands, and included evaluator-guided practice with confirmatory feedback to support task comprehension. Using the same procedure as in the spatial working memory task, the analysis for the current data revealed satisfactory internal consistency (α = 0.72).

The numerical working memory task is an adaptation of the Working Memory-Counting task (Case et al. 1982) designed to assess the participant’s ability to count and remember quantities using a visual and numerical recall format. In this task, participants are shown a series of cards displaying a mix of blue and yellow dots. They must count the yellow dots on each card and remember their quantities as the cards increase from two up to five in sequence. After viewing each card, participants select the corresponding number of yellow dots from a list of numbers (0 to 9) displayed at the bottom of the screen. Initially, participants practice with the evaluator by counting dots on one card and then another and confirming their counts before proceeding to recall sequences starting from two cards and increasing to three, four, and five. The evaluator’s role includes instructing the child through the task, ensuring understanding and correct performance, and providing feedback such as “Great job, now move on to the next one!” to encourage accurate recall and progression in the task. This task measures the capacity of numerical working memory by progressively challenging the participant’s ability to retain and sequence numerical information accurately. Using the same procedure as in the spatial working memory task, the analysis revealed a satisfactory internal consistency (α = 0.72).

#### 2.2.6. Math Anxiety

Mathematics anxiety was assessed using the Child Mathematics Anxiety Questionnaire—Revised (CMAQ-R; Ramirez et al. 2016), adapted for Spanish-speaking populations (Guzmán et al. 2021, 2023). The CMAQ-R is a validated, individually administered instrument designed specifically for young children in first and second grade. It consists of 16 items describing common mathematics-related situations, evenly split between classroom contexts (e.g., being called to the board) and specific mathematical problem-solving tasks (e.g., completing an addition problem). Children indicate their level of anxiety for each situation by selecting from a five-point pictorial Likert scale featuring cartoon faces that range from “not nervous at all” to “very, very nervous.” This visual scale has been demonstrated to be developmentally appropriate and easily interpretable by young children. Responses are scored from 1 to 5, with total scores ranging from 16 to 80, where higher scores reflect greater levels of mathematics anxiety. In the current study, the CMAQ-R showed strong internal consistency, with a Cronbach’s alpha of 0.86.

### 2.3. Procedure

Testing was divided into three separate sessions within a one-month period to minimize fatigue, as children were also assessed on additional tasks not included in the present study. All assessments were conducted in the spring of the academic year at the children’s respective schools, in quiet rooms designated by school principals or teachers. The first session included an auditory lexical decision task. The second session comprised the Child Mathematics Anxiety Questionnaire–Revised (CMAQ-R), spatial and numerical working memory tasks, and a semantic fluency task. The third session featured the Test of Early Mathematics Ability, Third Edition (TEMA-3). Research assistants, trained and verified for reliability by lead project staff prior to data collection, administered assessments individually using standardized instructions from a printed manual. Computer-based tasks were delivered via a touchscreen laptop running Unity 2019.4.20f1, with a consistent task order across all participants.

### 2.4. Analyses

Missing data were handled using multiple imputation by chained equations (MICE; Van Buuren and Groothuis-Oudshoorn 2011), implemented in the mice package in R. We used predictive mean matching (PMM), a robust method for ordinal and continuous data, with five imputations, 50 iterations, and a fixed random seed for reproducibility.

Mathematics performance was conceptualized as a latent variable defined by three observed indicators: number facts, written calculations, and calculation concepts. To investigate the relationships among vocabulary (both general and math-specific), working memory, math anxiety, and math performance, we employed structural equation modelling (SEM). This approach allowed for the simultaneous estimation of direct and indirect effects of cognitive (vocabulary and working memory) and affective (math anxiety) predictors on math performance.

Working memory was modelled as a latent construct composed of three indicators: spatial working memory (forward and backward span) and numerical working memory, reflecting its multidimensional nature as supported by previous research (e.g., Bull and Lee 2014). Math anxiety was also included as a latent mediator, consistent with its theoretical and psychometric structure, and was split into two distinct but correlated components: anxiety toward Explicit Numerical Situations (ENS) and General Classroom Situations (GCS), each defined by multiple items from the Child Mathematics Anxiety Questionnaire—Revised (CMAQ-R; Ramirez et al. 2016).

General vocabulary was modelled as a latent variable composed of two verbal fluency tasks, while math-specific vocabulary was included as a manifest variable. The model allowed for the examination of both independent and sequential mediating effects of working memory and the two math anxiety components on the relationship between vocabulary and math performance.

Given that math anxiety items were measured on ordinal Likert-type scales and the overall model included a mix of ordinal and continuous variables, the Weighted Least Squares Mean and Variance adjusted (WLSMV) estimator was used. WLSMV is appropriate for ordinal indicators and does not assume multivariate normality.

Model fit was evaluated using multiple indices: Comparative Fit Index (CFI), Tucker–Lewis Index (TLI), Root Mean Square Error of Approximation (RMSEA), Standardized Root Mean Square Residual (SRMR). In line with the guidelines proposed by Hu and Bentler (1999) and (Steiger 2007), model fit was considered acceptable if RMSEA ≤ 0.07 (with a 90% confidence interval not exceeding 0.06), SRMR ≤ 0.08, and both CFI and TLI ≥ 0.95.

## 3. Results

R Software version 4.3.1 (R Core Team 2023) was employed to produce all analyses. To ensure transparency in the measurement models, standardized factor loadings and McDonald’s omega for each latent construct (Working Memory, Math Performance, General Vocabulary, Explicit Numerical Situations, and General Classroom Situations) are reported in Appendix A. Inter-indicator correlations for all measures are provided in Appendix B. Table 1 presents the means, standard deviations, and correlations among all measured variables. Overall, the pattern of correlations aligns with theoretical expectations. Both general and math-specific vocabulary were positively associated with working memory and math performance, suggesting that richer lexical knowledge supports both cognitive processing and achievement in mathematics. Conversely, math anxiety was negatively related to working memory and all other variables, indicating its potential role as an affective barrier to mathematical learning. Of note, working memory demonstrated the strongest association with math performance among the predictors, underscoring its central role in supporting mathematical competence.

**Figure 1:**
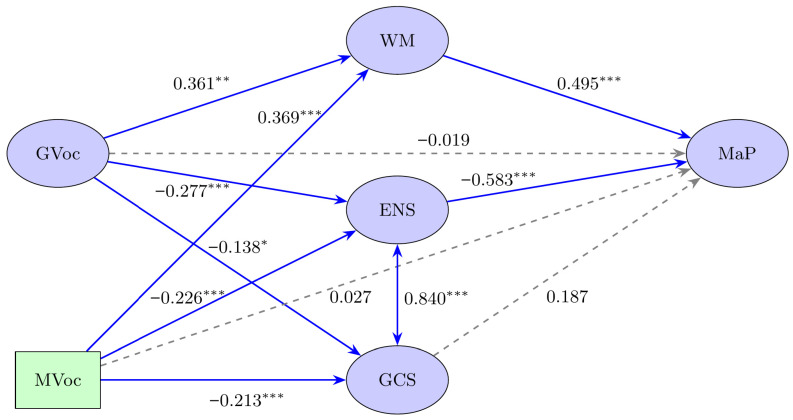
Structural Equation Model (SEM) of general vocabulary (GVoc), math vocabulary (MVoc), working memory (WM), math anxiety [Explicit numerical situations (ENS), general classroom situations (GCS)], and math performance (MaP). Significance: * *p* < 0.05, ** *p* < 0.01, *** *p* < 0.001.

The proposed structural equation model was tested to examine whether working memory and math anxiety mediate the associations between vocabulary (general and math-specific) and math performance. The model demonstrated an acceptable fit to the data, χ^2^(264) = 501.75, *p* < .001; CFI = 0.936; TLI = 0.933; RMSEA = 0.044 [90% CI: 0.038, 0.050]; SRMR = 0.057, based on recommended thresholds (Hu and Bentler 1999; Steiger 2007). Before examining the structural relationships, the measurement models for the latent constructs were validated, confirming acceptable reliability (see Appendix A). Standardized path coefficients are presented in Table 2. General and math-specific vocabulary significantly predicted working memory, which in turn predicted math performance. Both types of vocabulary were negatively associated with math anxiety, measured as two latent factors: Explicit numerical situations (ENS) and General classroom situations (GCS). Among the two, only ENS was significantly and negatively associated with math performance. Indirect effects indicated that vocabulary exerted its influence on math performance through both working memory and math anxiety (particularly ENS), supporting a dual mediation model. The model explained 52% of the variance in math performance (R^2^ = 0.52), indicating a strong predictive capacity. See Figure 1.

## 4. Discussion

The present study examined how general and math-specific vocabulary contribute to individual differences in math performance among Chilean second-grade children, and whether these effects are mediated by working memory and math anxiety. Using structural equation modelling, we tested a dual-pathway model in which vocabulary was posited to influence math performance indirectly through two distinct mechanisms: a cognitive pathway via working memory and an affective pathway via math anxiety. In modelling math anxiety, we distinguished between two dimensions: explicit numerical situations (ENS) and general classroom situations (GCS), as assessed through the CMAQ-R (Ramirez et al. 2016; Guzmán et al. 2021).

The findings revealed that both general and math-specific vocabulary were significantly associated with better math performance, but these effects were fully mediated through working memory and ENS-type math anxiety. Notably, GCS-related anxiety was not significantly associated with math performance, highlighting the importance of situational specificity when assessing emotional factors in early mathematics learning. The final model accounted for 52% of the variance in math performance, indicating strong explanatory power. These results support a dual mediation model, underscoring that cognitive and affective factors work in tandem to link linguistic knowledge to early numeracy outcomes. In the following sections, we discuss the findings in relation to our research questions and hypotheses and reflect on their theoretical and practical implications for early math education.

### 4.1. What Is the Relationship Between Vocabulary (General and Math-Specific) Math Anxiety, and Math Performance in Second-Grade Students?

Consistent with Hypotheses a and b, both general and math-specific vocabulary were significantly and positively correlated with math performance. Conversely, math anxiety, particularly in explicit numerical situations (ENS), was negatively associated with math outcomes. These findings replicate and extend prior research highlighting the foundational role of language in mathematical development (LeFevre et al. 2010; Purpura and Logan 2015; Riccomini et al. 2015). General vocabulary supports the comprehension of instructions and verbally mediated tasks, whereas math-specific vocabulary provides the semantic tools necessary to represent and manipulate formal mathematical concepts (Bleses et al. 2023; Lin et al. 2021).

Importantly, both vocabulary types were also negatively associated with math anxiety, suggesting that children with stronger lexical representations, particularly in mathematics, may feel more competent and thus experience less anxiety when faced with numerical tasks. This aligns with theoretical models proposing that linguistic fluency mitigates affective responses by reducing ambiguity and perceived cognitive load during math problem-solving (Carey et al. 2017; Ramirez et al. 2018). This pattern also suggests that vocabulary-based interventions, especially those that strengthen math-specific language, may have the potential to reduce children’s anxiety toward mathematical tasks, providing a promising avenue for early educational support.

In contrast to previous studies that assessed math anxiety as a global construct, our model differentiated between anxiety in explicit numerical situations (ENS) and in general classroom settings (GCS). Only ENS was significantly associated with math performance, reinforcing the idea that task-specific emotional responses, rather than more diffuse classroom anxiety, have a stronger impact on actual numerical performance (Maloney and Beilock 2012; Ramirez et al. 2013). This distinction underscores the value of context-sensitive assessment tools in identifying the specific dimensions of anxiety that interfere with children’s mathematical achievement. This pattern converges with recent evidence indicating that calculation-specific anxiety, rather than general classroom-based anxiety, uniquely predicts mathematical performance when both dimensions are entered into the same structural model (Lukowski et al. 2019).

### 4.2. Do Working Memory and Math Anxiety Mediate the Relationship Between Vocabulary and Math Performance?

Supporting hypotheses c and d, our model revealed that both working memory and math anxiety in explicit numerical situations (ENS) mediated the effects of general and math-specific vocabulary on math performance, constituting a dual pathway from language to mathematical outcomes. General and math-specific vocabulary were each positively associated with working memory, which in turn significantly predicted math performance.

These findings are consistent with longitudinal and meta-analytic evidence demonstrating a reciprocal relationship between vocabulary and working memory skills across development (Bruce and Bell 2022; Shokrkon and Nicoladis 2022). This interplay likely reflects both the role of vocabulary in scaffolding working memory processes and the importance of executive function in learning and integrating new lexical items. as well as studies showing that working memory enables the application of linguistic knowledge in cognitively demanding mathematical tasks (Hornburg et al. 2024; Peng et al. 2016).

The observed mediation via ENS is particularly noteworthy. Both vocabulary dimensions were negatively associated with ENS, which in turn was negatively associated with math performance, supporting the idea that linguistic proficiency may buffer against affective disruption in math contexts. This result aligns with theoretical accounts proposing that vocabulary depth enhances conceptual clarity and confidence, reducing the cognitive and emotional strain often triggered by numerically demanding tasks (Beilock and Ramirez 2011; Carey et al. 2017).

Importantly, while both ENS and working memory served as significant mediators, they appeared to operate through partially distinct mechanisms. Working memory represents a domain-general cognitive resource that supports the active maintenance and manipulation of information, whereas ENS captures a domain-specific emotional response that can interfere with these processes by consuming attentional and cognitive resources (Ashcraft and Kirk 2001). Neurocognitive research further supports this interpretation, showing that anxiety-related activation can reduce efficiency in brain networks responsible for working memory and mathematical reasoning (Pelegrina et al. 2020; Rose et al. 2023; Yu 2023). Thus, these two mediators jointly shape how vocabulary is translated into mathematical performance, emphasizing the need for integrated models that consider both affective and cognitive pathways.

### 4.3. Are the Indirect Effects Stronger for Math-Specific Vocabulary than for General Vocabulary?

In line with hypothesis e, math-specific vocabulary demonstrated slightly stronger total and indirect effects on math performance than general vocabulary. While both types of vocabulary contributed meaningfully to math achievement through working memory and ENS, the effects of math-specific vocabulary were more directly aligned with the conceptual content and terminological demands of mathematics. This is consistent with meta-analytic findings that domain-specific vocabulary predicts mathematical learning over and above general language skills (Lin et al. 2021; Peng and Lin 2020).

These findings underscore the distinct cognitive demands of mathematical reasoning. Unlike general vocabulary, which supports broader linguistic comprehension, math-specific vocabulary enables learners to internalize and retrieve precise conceptual representations (e.g., “quotient,” “sum”) that directly map onto the structure of formal math problems (Toll and Van Luit 2014). As a result, math-specific vocabulary may more effectively engage working memory during numerical problem solving, enhancing task fluency and reducing ambiguity. Furthermore, by equipping students with more targeted verbal tools, math-specific vocabulary may also reduce anxiety in math-specific contexts (ENS), functioning as both a cognitive scaffold and an emotional buffer. This dual influence strengthens the translational implications of our findings: interventions designed to enhance math vocabulary may not only support cognitive outcomes but also mitigate affective barriers to math learning.

### 4.4. Limitations and Directions for Future Research

While our study provides novel insights into the dual cognitive and affective pathways linking language to mathematical performance in early learners, some limitations should be acknowledged.

First, the cross-sectional nature of our design constrains causal interpretation. Although the proposed mediational pathways are theoretically and empirically grounded, longitudinal studies are necessary to trace developmental trajectories over time and to assess the directionality and potential bidirectionality of the relationships between vocabulary, working memory, anxiety, and math achievement.

Second, although we differentiated between math anxiety in explicit numerical situations (ENS) and general classroom situations (GCS), only ENS was significantly related to performance. This specificity supports the notion that anxiety effects are context-dependent; however, future work should further explore the dimensionality and developmental course of math anxiety in young children.

Third, there is potential overlap between the semantic fluency task used for general vocabulary and working memory, as verbal fluency tasks may engage working memory processes (Unsworth et al. 2013). This could inflate the relationship between general vocabulary and working memory, warranting caution in interpreting their mediation effects.

Fourth, another potential limitation is the fixed order of administration, where the Child Mathematics Anxiety Questionnaire–Revised (CMAQ-R) preceded the working memory tasks and TEMA-3, which may have introduced priming effects despite the one-month interval between sessions (Ramirez et al. 2016). Future research should counterbalance task order to mitigate this risk.

Finally, our findings are based on a Chilean sample, contributing valuable data from a population underrepresented in cognitive and educational research. While this adds important cultural and linguistic diversity to the literature, replication across other languages, orthographies, and educational systems is essential to assess the generalisability of the proposed model. Socioeconomic, curricular, and pedagogical variables may moderate the relationships observed here and should be systematically explored in future cross-cultural studies.

## 5. Conclusions

This study contributes to a growing body of evidence underscoring the intertwined roles of language, cognition, and affect in early mathematical development. By testing a dual-pathway mediation model, we demonstrated that both general and math-specific vocabulary influence math performance indirectly through working memory and math anxiety, particularly in explicit numerical situations. These findings provide strong support for theoretical frameworks that emphasize the importance of both cognitive resources and emotional states in academic achievement. Crucially, our results highlight that math-specific vocabulary exerts slightly stronger effects on math outcomes than general vocabulary, reinforcing the unique value of domain-specific language in supporting children’s conceptual understanding and task execution. Moreover, by differentiating between distinct forms of math anxiety, we show that not all affective responses exert equivalent effects on performance. The finding that only anxiety in explicit numerical contexts predicted achievement underscores the need for nuanced assessment tools and targeted interventions. Together, these insights offer practical implications for educational practice. Interventions that strengthen children’s vocabulary, particularly their command of mathematical language, may yield both cognitive and emotional benefits, enhancing working memory performance while also reducing math-specific anxiety. Addressing both pathways simultaneously may be especially effective in supporting learners at risk of academic difficulties. Future research should continue to explore how language, memory, and emotion interact across development and across diverse sociolinguistic contexts.

## Figures and Tables

**Table 1:** Descriptive statistics and correlations for all measured variables.

Measured Variable	M	SD	1	2	3	4	5
1. General Vocabulary (GVoc)	12.18	3.64	—				
2. Math Vocabulary (MVoc)	30.51	4.45	.19 ***	—			
3. Working Memory (WM)	3.01	1.23	.16 **	.26 ***	—		
4. Math Anxiety (MA)	2.35	0.68	–.14 **	–.19 ***	–.17 ***	—	
5. Math Performance (MaP)	2.33	1.62	.25 ***	.28 ***	.41 ***	–.27 ***	—

Note. *** *p* < .001, ** *p* < .01.

**Table 2:** Standardized direct, indirect, and total effects from the structural equation model.

Predictor	Outcome	Direct Effect β	Indirect Effect β	Total Effect β	*p*
General Vocabulary	Math Performance	–.019	.314	.295	<.001
Math Vocabulary	Math Performance	.027	.274	.301	<.001
Working Memory	Math Performance	.495	—	—	<.001
ENS	Math Performance	–.583	—	—	<.001
GCS	Math Performance	.187	—	—	.167
General Vocabulary	Working Memory	.361	—	—	.002
Math Vocabulary	Working Memory	.369	—	—	<.001
General Vocabulary	ENS	–.277	—	—	<.001
Math Vocabulary	ENS	–.226	—	—	<.001
General Vocabulary	GCS	–.138	—	—	.044
Math Vocabulary	GCS	–.213	—	—	<.001

Note. ENS = Explicit numerical situations (math anxiety); GCS = General classroom situations (math anxiety). Indirect effects are calculated through mediators (working memory, ENS, GCS).

## Data Availability

The data used in this study is available at https://osf.io/vcjhy/files/osfstorage/6862def70c34746ed31288b1 (accessed on 14 April 2025).

## Appendix A. Measurement Model Details

**Table A1:** Standardized factor loadings, mean standardized loading, and McDonald’s omega (ω) for each latent construct in the structural equation model.

Latent Variable	Number of Indicators	Indicators (Standardized Loadings)	Mean Standardized Loading	McDonald’s Omega (ω)
Working Memory (WM)	3	WM_forward (0.481), WM_backward (0.661), WM_numerical (0.547)	0.563	0.615
Math Performance (MaP)	3	NF (0.786), Calculation (0.858), Concepts (0.818)	0.821	0.861
General Vocabulary (GVoc)	2	GVoc1_fluency (0.589), GVoc2_fluency (0.712)	0.651	0.724
Explicit Numerical Situations (ENS)	9	MA1 (0.428), MA3 (0.711), MA4 (0.499), MA5 (0.516), MA7 (0.507), MA9 (0.690), MA13 (0.647), MA14 (0.660), MA16 (0.748)	0.601	0.834
General Classroom Situations (GCS)	7	MA2 (0.622), MA6 (0.595), MA8 (0.560), MA10 (0.642), MA11 (0.634), MA12 (0.552), MA15 (0.602)	0.601	0.797

Note. NF = Number facts; MA = Math anxiety.

## Appendix B. Inter-Indicator Correlations

**Table A2:** Correlations among the 24 indicators used in the structural equation model.

	WMf	WMb	WMn	NF	Calc	Conc	GV1	GV2	MA1	MA3	MA4	MA5	MA7	MA9	MA13	MA14	MA16	MA2	MA6	MA8	MA10	MA11	MA12	MA15
WM_f	1	0.46	0.24	0.21	0.23	0.19	0	0.1	0	−0.16	−0.15	0.02	−0.03	−0.17	−0.06	−0.11	−0.16	−0.03	−0.05	−0.03	−0.15	−0.1	−0.03	−0.15
WM_b	0.46	1	0.29	0.34	0.33	0.28	0.13	0.15	−0.15	−0.22	−0.07	−0.07	−0.05	−0.26	−0.15	−0.22	−0.21	−0.11	−0.14	−0.06	−0.1	−0.06	−0.01	−0.08
WM_n	0.24	0.29	1	0.27	0.31	0.29	0.11	0.15	−0.1	−0.13	−0.09	0	−0.05	−0.16	−0.12	−0.16	−0.18	−0.1	−0.15	−0.13	−0.11	−0.06	−0.06	−0.07
NF	0.21	0.34	0.27	1	0.68	0.64	0.16	0.18	−0.19	−0.27	−0.12	−0.27	−0.12	−0.31	−0.27	−0.23	−0.33	−0.19	−0.06	−0.16	−0.32	−0.14	−0.12	−0.23
Calc	0.23	0.33	0.31	0.68	1	0.69	0.22	0.2	−0.23	−0.34	−0.2	−0.15	−0.14	−0.33	−0.31	−0.27	−0.36	−0.21	−0.14	−0.17	−0.31	−0.17	−0.19	−0.23
Conc	0.19	0.28	0.29	0.64	0.69	1	0.21	0.21	−0.16	−0.33	−0.13	−0.15	−0.13	−0.33	−0.26	−0.16	−0.33	−0.15	−0.09	−0.16	−0.2	−0.18	−0.17	−0.19
GV1	0	0.13	0.11	0.16	0.22	0.21	1	0.57	−0.07	−0.16	−0.15	0.05	−0.02	−0.24	−0.05	−0.06	−0.17	−0.04	−0.07	−0.07	−0.04	−0.1	−0.04	−0.13
GV2	0.1	0.15	0.15	0.18	0.2	0.21	0.57	1	−0.04	−0.18	−0.11	0.03	−0.15	−0.21	−0.11	−0.15	−0.14	0	−0.11	−0.1	−0.02	−0.15	−0.02	−0.11
MA1	0	−0.15	−0.1	−0.19	−0.23	−0.16	−0.07	−0.04	1	0.35	0.12	0.25	0.29	0.21	0.39	0.23	0.31	0.13	0.22	0.22	0.26	0.26	0.16	0.11
MA3	−0.16	−0.22	−0.13	−0.27	−0.34	−0.33	−0.16	−0.18	0.35	1	0.29	0.39	0.41	0.44	0.49	0.45	0.48	0.32	0.27	0.4	0.38	0.35	0.32	0.39
MA4	−0.15	−0.07	−0.09	−0.12	−0.2	−0.13	−0.15	−0.11	0.12	0.29	1	0.18	0.23	0.36	0.24	0.48	0.29	0.3	0.36	0.21	0.27	0.28	0.34	0.34
MA5	0.02	−0.07	0	−0.27	−0.15	−0.15	0.05	0.03	0.25	0.39	0.18	1	0.18	0.38	0.35	0.32	0.37	0.31	0.14	0.31	0.31	0.25	0.25	0.35
MA7	−0.03	−0.05	−0.05	−0.12	−0.14	−0.13	−0.02	−0.15	0.29	0.41	0.23	0.18	1	0.3	0.38	0.41	0.33	0.26	0.23	0.24	0.29	0.38	0.19	0.12
MA9	−0.17	−0.26	−0.16	−0.31	−0.33	−0.33	−0.24	−0.21	0.21	0.44	0.36	0.38	0.3	1	0.44	0.45	0.59	0.35	0.31	0.26	0.35	0.36	0.28	0.32
MA13	−0.06	−0.15	−0.12	−0.27	−0.31	−0.26	−0.05	−0.11	0.39	0.49	0.24	0.35	0.38	0.44	1	0.37	0.47	0.35	0.3	0.34	0.32	0.34	0.36	0.27
MA14	−0.11	−0.22	−0.16	−0.23	−0.27	−0.16	−0.06	−0.15	0.23	0.45	0.48	0.32	0.41	0.45	0.37	1	0.47	0.39	0.38	0.42	0.37	0.38	0.27	0.32
MA16	−0.16	−0.21	−0.18	−0.33	−0.36	−0.33	−0.17	−0.14	0.31	0.48	0.29	0.37	0.33	0.59	0.47	0.47	1	0.35	0.36	0.34	0.45	0.41	0.27	0.42
MA2	−0.03	−0.11	−0.1	−0.19	−0.21	−0.15	−0.04	0	0.13	0.32	0.3	0.31	0.26	0.35	0.35	0.39	0.35	1	0.4	0.34	0.47	0.35	0.4	0.35
MA6	−0.05	−0.14	−0.15	−0.06	−0.14	−0.09	−0.07	−0.11	0.22	0.27	0.36	0.14	0.23	0.31	0.3	0.38	0.36	0.4	1	0.42	0.36	0.46	0.34	0.4
MA8	−0.03	−0.06	−0.13	−0.16	−0.17	−0.16	−0.07	−0.1	0.22	0.4	0.21	0.31	0.24	0.26	0.34	0.42	0.34	0.34	0.42	1	0.18	0.25	0.28	0.36
MA10	−0.15	−0.1	−0.11	−0.32	−0.31	−0.2	−0.04	−0.02	0.26	0.38	0.27	0.31	0.29	0.35	0.32	0.37	0.45	0.47	0.36	0.18	1	0.42	0.39	0.34
MA11	−0.1	−0.06	−0.06	−0.14	−0.17	−0.18	−0.1	−0.15	0.26	0.35	0.28	0.25	0.38	0.36	0.34	0.38	0.41	0.35	0.46	0.25	0.42	1	0.35	0.32
MA12	−0.03	−0.01	−0.06	−0.12	−0.19	−0.17	−0.04	−0.02	0.16	0.32	0.34	0.25	0.19	0.28	0.36	0.27	0.27	0.4	0.34	0.28	0.39	0.35	1	0.33
MA15	−0.15	−0.08	−0.07	−0.23	−0.23	−0.19	−0.13	−0.11	0.11	0.39	0.34	0.35	0.12	0.32	0.27	0.32	0.42	0.35	0.4	0.36	0.34	0.32	0.33	1

Note. WM_f = Working memory (forward); WM_b = Working memory (backward); WM_n = Working memory (numerical); NF = Number facts; Calc = Calculation; Conc = Concepts; GV =General vocabulary; MA = Math anxiety.
